# Quality of life after total laryngectomy: a retrospective study

**DOI:** 10.1192/j.eurpsy.2025.1792

**Published:** 2025-08-26

**Authors:** N. Sghaier, R. Ben Soussia, W. Zaier, W. Bouali, F. Youzbechi, H. Ben Garouia, L. Zarrouk

**Affiliations:** 1 Psychiatry Departement, Ibn Jazzar Hospital, Kairouan; 2 Psychiatry Departement, Tahar Sfar Hospital, Mahdia, Tunisia

## Abstract

**Introduction:**

Total laryngectomy, as a major surgical intervention, leads to significant changes both physically and psychologically. The observed alterations include communication difficulties, adjustments to new breathing methods, and often a lengthy and complex rehabilitation process. These changes can deeply impact social interactions, autonomy, and the emotional well-being of the individuals affected.

**Objectives:**

To assess quality of life in patients who have undergone total laryngectomy, and to investigate the factors associated with its impairment.

**Methods:**

This was a cross-sectional descriptive study with an analytical aim conducted over a period of 6 months, from December 2021 to June 2022, involving patients followed for laryngeal cancer who had undergone total laryngectomy at the Otorhinolaryngology department of Tahar Sfar Mahdia Hospital. Quality of life was assessed using the SF-36 in its validated Arabic version. Referring to Lean’s threshold value, it is accepted that a mean score < 66.7 indicates an impairment in quality of life.

**Results:**

A total of 40 patients participated in the study. The mean age was 62±9 years, and the population was 100% male. Quality of life assessment revealed that 87% of patients had scores below 66.7, attesting to impaired quality of life. A study of the mean scores per dimension on the SF36 revealed the following rates of impairment, in descending order: 100% for physical pain (D3), 97% for psychological health (D8), 95% for life and relationships with others (D6), 87% for vitality (D5) and limitations due to psychological state (D7), 72% for perceived health (D4), 55% for physical activity (D1) and 22% for limitations due to physical state (D2). In univariate analysis, rural origin (p=0.015), low socio-economic level (p=0.023), the existence of discomfort with eating (p=0.002), the existence of pain (p=0.008) and long hospital stay (p=0.056) were associated with impaired quality of life in our study population.

**Conclusions:**

It is crucial to recognize and address these challenges holistically. Multidisciplinary approaches, including voice rehabilitation, psychological support, and social assistance, play an essential role in improving the quality of life for total laryngectomy patients.

**Disclosure of Interest:**

None Declared

